# Socioeconomic differences in children’s victimization to maternal and paternal violence: a register-based study

**DOI:** 10.1177/14034948231180670

**Published:** 2023-08-17

**Authors:** Noora Ellonen, Joonas Pitkänen, Mikko Aaltonen, Hanna Remes, Pekka Martikainen

**Affiliations:** 1 Faculty of Social Science, Tampere University, Finland; 2 University of Helsinki, Finland; 3 Law School, University of Eastern Finland, Finland

**Keywords:** Children, parents, violence, victimization, socioeconomic position, register data

## Abstract

**Aims:**

To explore the potential of administrative data in assessment of the association between parental socioeconomic position (SEP) and children’s violent victimization by biological parents.

**Methods:**

A longitudinal register-linkage study based on child–mother and child–father data, including all children born in Finland between 1991 and 2017. The data included 1,535,428 children, 796,335 biological mothers, and 775,966 fathers. We used logistic regression with person-years as observations and cluster-robust standard errors to predict children’s violent victimization in 2009–2018 and assessed effect modification by child’s age and gender.

**Results:**

For the SEP indicators, lower maternal education (adjusted odds ratio (OR) 2.90, secondary education OR 1.99) and lower paternal education (OR 2.24, secondary education OR 1.59) were risk factors for violent victimization. Parental social assistance receipt (OR 2.4) and non-employment (OR 1.8–1.9) increased the risk of victimization to maternal and paternal violence. Income was associated with victimization in a gradient-like manner, with ORs ranging from 1.14 to 1.98 among mothers and from 1.29 to 2.56 among fathers. Children with low parental SEP were at the highest risk of parental violence, particularly paternal violence, at ages 3−8 years.

**Conclusions:**

**All indicators of low SEP increased the risk of children experiencing both maternal and paternal physical violence, especially at ages 3–8 years. Longitudinal register data—because of large samples, no nonresponse or self-report bias, and the possibility to analyze violence committed by mother and father and age-groups separately—have great potential for comprehensive research on the risk factors of parental violence that are difficult to reliably assess with other types of data**.

## Introduction

Much research has shown that low socioeconomic position (SEP), indicated by, for example, parental unemployment, poverty, and low education, is associated with a higher risk of children experiencing violence at home [1,2]. However, in Nordic countries the findings are not consistent. For example, the findings from two recent national Finnish surveys based on parental reports on children’s experiences of violence [3-5] and a Norwegian study that examined violence against children at home during COVID-19 lockdowns [6] have suggested no or minimal association between low parental SEP and children’s experiences of violence.

According to recent literature reviews [2,7,8] this variation is likely to be due to the limitations of survey data. Although important for descriptions of victimization trends, survey data may suffer from nonresponse biases, which lead to sample selection, and recall and other response biases, particularly when dealing with sensitive topics such as violence [9]. Moreover, individuals may have different propensities for what they deem violence and to what extent they want to disclose it. Children and adolescents may also be unable to accurately report their parent’s SEP [10].

Because of the large samples, no nonresponse or self-report bias, and the longitudinal nature of the data, administrative register data have recently been suggested as a valuable but underused resource for improving our understanding of the etiology of child maltreatment [8,11]. The potential lies particularly in Nordic register data: a wide range of government-maintained, nationwide, public registries that include individual-level data that can be linked through personal identification numbers to allow unique coverage [12]. Linkages enable a wider exploration of child maltreatment indicators, different risk and protective factors, and outcomes for children who experience violence. However, the association between SEP and parental violence toward their children has not yet been studied based on Nordic administrative register data.

In this paper, we explore the potential of routinely collected administrative register data to assess the associations of parental education, non-employment, income, and economic hardship with police-reported parental violence against children. We also examine whether the children’s age may modify the associations between socioeconomic measures and the risk of violence: such a hypothesis was warranted based on descriptive survey studies that show that parental violence against children varies according to the child’s age. In Finland, the highest rates of violence have been observed among toddlers and teenagers [13], while in the United States, the rate of parental violence increases somewhat linearly by the child’s age [14]. Prior to this study, we knew little about the social backgrounds of offenders in police-reported cases of family violence, or socioeconomic differences in the age patterns of children’s violent victimization by parents. Therefore, our study will provide new insights into the literature on socioeconomic differences in children’s violent victimization at home.

## Methods

### Data

This study was based on administrative register data on all 0–55-year-old women residing in Finland between 2000 and 2015. The dataset contains individual identification numbers, enabling linkages to all the women’s children born until 2017, and the children’s biological fathers. We constructed separate datasets for children and their mothers, and for children and their fathers. In the child–mother data, we included all children born in Finland between 1991 and 2017 who had resided in Finland during at least one year between 2008 and 2017 (*N* = 1,578,007). Of these children, we excluded those whose mothers had not resided in Finland during the same years (*N* = 4,195), resulting in a final analytical sample of 1,573,812 children and 796,335 biological mothers. Similarly, in the child–father data, we excluded children whose fathers had not resided in Finland between 2008 and 2017 (*N* = 18,905) as well as those whose father could not be identified (*N* = 23,674), thus arriving at an analytical sample of 1,535,428 children and 775,966 fathers. We included all person-years during 2008–2017, when the children and their parents lived in Finland and the children were aged 0–17 in both the child–mother and child–father data.

Next, we linked all the parents and the children with the sociodemographic information collected by Statistics Finland’s annual population registers. The data were then linked with Statistics Finland’s domestic violence data for the years 2009–2018 to predict child’s victimization in a given year using indicators of parental SEP measured during the preceding year (2008–2017). Information on domestic violence was derived from the records of suspected offenses known to police, and included the identification numbers of the victims and perpetrators, the type of crime, and the year the crime took place. We only included violent crimes (as determined by penal codes for petty assault, assault, aggravated assault, and attempted homicides/murders/assaults/aggravated assaults) in our analysis. Over 80% of the cases were assaults. The data did not include crimes that resulted in death.

### Outcome

The outcome variable in this study was parental violence against children based on crimes reported to the police. The unit of observation was the child, and the experience of maternal and/or paternal violence was the outcome. Although these were suspected offenses, for simplicity, we refer to them here as violent crime. The outcome was 1 if a biological parent had been suspected of committing a violent crime against their child during any given year, and 0 otherwise. Hence, the variable measured the risk of being a victim of a violent crime committed by a biological parent during a calendar year.

### Parental socioeconomic position

We focused on four different indicators of parental SEP to cover its different dimensions and assess whether the association between SEP and violence depends on the operationalization of SEP. Although previous survey-based studies have suggested there is no association between parental SEP and children’s victimization at home in Finland, associations between other parent-related factors and the risk for violence have shown to be different between paternal and maternal violence [3,4]. Therefore, we conducted separate analyses of paternal and maternal violence, and adjusted our models for family characteristics, such as family size, parental age, and living arrangements. We measured education, receipt of social assistance, non-employment status, and income. The highest level of parental education was classified as either no secondary/secondary/any tertiary. All individuals who had received any social assistance in a given year were considered to be recipients of social assistance. The unemployed and those outside the labor force, but not studying or in training, were classified as non-employed, based on Statistics Finland’s information for the main type of economic activity during the whole year. Finally, income was based on information about a parent’s disposable personal income from the Finnish Tax Administration’s database. We calculated income quintiles for mothers and fathers separately for each calendar year. For each annual observation of parental violence, the information for socioeconomic variables was derived from the end of the preceding year (i.e., we predicted the current year’s crime by last year’s SEP).

### Covariates

We adjusted our analyses for several time-varying covariates, which could have confounded the association between parental SEP and the risk of parental violence. We included information about the child’s current living arrangements, classified as two-parent families (parents and child living in same household), single-parent families (parent and child in same household), and others. We also included information on the number of biological (maternal or paternal in the corresponding analyses) 0–17-year-old siblings (0/1/2/3/4/5+) in the analytical sample, regardless of whether they were living in the same household. Finally, we adjusted for parental age (under 25/25–34/35–44/45–54/55+), the parent’s country of birth (Finland/other), the child’s gender and age (0–2/3–5/6–8/9–11/12–14/15–17), and the calendar year in our analyses.

## Modeling strategy

We used person-years as observations and logistic regression with cluster-robust (with parent as the cluster) standard errors to measure socioeconomic differences in the children’s experiences of parental violence. All the SEP variables were examined in separate analyses. We fitted two models for each SEP variable: a crude model and a model adjusted for all covariates. We also conducted several analyses for effect modification. First, we ran an interaction analysis between child’s age and parental SEP variables, adjusting for the covariates, and then repeated this analysis stratified by child’s gender. Second, we ran an interaction analysis between child’s gender and parental SEP. The results of the interaction analyses are presented as average predicted probabilities holding covariates as observed.

Finally, as a sensitivity check and to enable a better comparison of our findings to those of survey studies, we combined information on violent victimization from all the person-years together and estimated socioeconomic differences in the odds of ever experiencing violent victimization by parents during the follow-up years 2009–2018. In these analyses, parental SEP and covariates were measured at the first observation in the data. Instead of including the child’s age and the calendar year as model covariates, child’s year of birth was adjusted for.

## Results

### Descriptive characteristics

In total, 3987 children (4192 person-year observations) experienced maternal violence, of which 50% were girls, and 8% were victims of maternal violence more than once. Of the 3098 mothers who perpetrated a crime, 26% did this more than once, and 21% had more than one victim. Experiences of paternal violence were more common than maternal violence. There were 6707 individual victims of paternal violence (7,118 person-year observations), of which 41% were girls, and 8% were victimized more than once. A fifth of the 5232 paternal perpetrators had more than one victim, and 28% had committed more than one violent crime.

**Figure 1.**
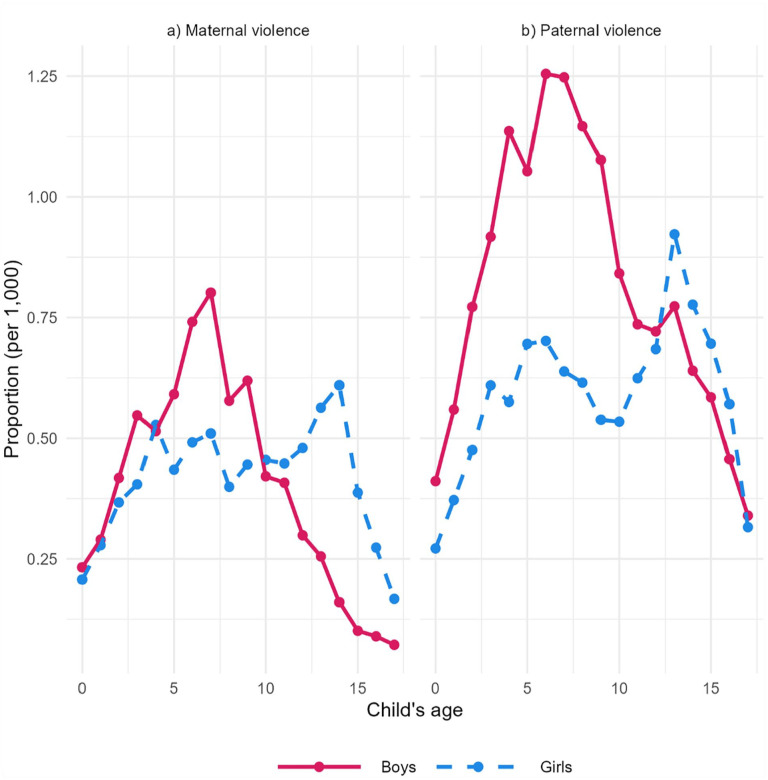
Proportion of violent victimizations per 1000 person-years by mothers and fathers according to child’s age, and child’s gender.

Figure 1 shows the proportion (per 1000 person-years) of being a victim of parental violence by child’s gender and age. Note that we used person-years as observations, and individuals were not omitted from the study after their first victimization. Therefore, children who had experienced violence several times may have contributed data to several age categories. The lowest risk for victimization was among the youngest and oldest children. Boys were more likely to experience violence from their fathers than from their mothers, especially when the boys were aged 4–9. The risk of victimization among boys increased by age, peaking at 7–8 years, and decreasing afterwards. Among girls, the risk factors of victimization were relatively similar, regardless of the parent’s gender. In contrast to boys, however, the highest risk of victimization among girls was in mid-adolescence, at the age of 14.

Table I shows the distributions of the SEP variables and covariates in the studied population, including the proportion (with an event) of child victimization. Across the SEP variable categories, the proportions were quite low. However, there were clear differences. Violent victimization was around six times more common among the children of mothers without secondary education when compared to the children of mothers with tertiary education, and around three times more common among the children whose fathers did not have secondary education when compared to the children whose fathers had tertiary education. The proportion of victimization among children of non-employed parents was three times higher than that of employed parents. An income gradient with the highest risk of victimization among those in the lowest quintile was observed.

**Table I.** Distribution of parental socioeconomic position (SEP) and sociodemographic characteristics, and prevalence (per 1000 person-years) of children’s violent victimizations in the study populations of child–mother and child–father dyads.

	Mothers			Fathers		
Parental SEP	Total person- years	Total, %	Prevalence, ‰	Total person- years	Total, %	Prevalence, ‰
Education						
No secondary	1,127,195	10.9	1.1	1,581,421	15.7	1.3
Secondary	4,171,428	40.2	0.5	4,799,761	47.5	0.7
Any tertiary	5,075,418	48.9	0.2	3,723,612	36.8	0.4
Social assistance receipt						
No	9,698,690	93.5	0.3	9,499,183	94	0.6
Yes	675,351	6.5	1.8	605,611	6	2.4
Non-employed						
No	8,690,037	83.8	0.3	9,298,233	92	0.6
Yes	1,684,004	16.2	0.9	806,561	8	1.8
Income quintile						
Lowest	1,949,774	18.8	0.5	1,873,141	18.5	1.3
2^nd^	2,013,831	19.4	0.5	1,980,231	19.6	0.8
3^rd^	2,025,560	19.5	0.4	2,049,380	20.3	0.6
4^th^	2,160,215	20.8	0.3	2,090,884	20.7	0.5
Highest	2,224,661	21.4	0.3	2,111,158	20.9	0.4
Covariates						
Living arrangements						
Two-parent (with index child)	8,342,917	80.4	0.3	7,824,731	77.4	0.5
Single parent (with index child)	1,569,244	15.1	1.1	218,437	2.2	2.6
Other	461,880	4.5	0.7	2,061,626	20.4	1.2
Parental age						
under 25	260,025	2.5	0.8	119,888	1.2	1.2
25–34	2,923,299	28.2	0.5	2,098,519	20.8	0.8
35–44	4,930,724	47.5	0.4	4,586,933	45.4	0.7
45–54	2,130,115	20.5	0.3	2,832,675	28	0.6
55+	129,878	1.3	0.1	466,779	4.6	0.7
Parent’s country of birth						
Finland	9,653,147	93.1	0.3	9,391,666.0	92.9	0.6
Other	720,894	6.9	1.3	713,128.0	7.1	1.7
Number of 0–17-year-old biological siblings in the analytical sample						
0	2,416,074	23.3	0.4	2,373,134	23.5	0.6
1	4,356,046	42	0.4	4,230,488	41.9	0.6
2	2,262,039	21.8	0.4	2,189,517	21.7	0.7
3	731,728	7.1	0.6	714,764	7.1	1.1
4	257,250	2.5	0.8	251,590	2.5	1.2
5 or more	350,904	3.4	0.4	345,301	3.4	0.9
Child’s gender						
Male	5,301,710	51.1	0.4	5,167,277	51.1	0.8
Female	5,072,331	48.9	0.4	4,937,517	48.9	0.6
Child’s age						
0–2	1,737,632	16.7	0.3	1,695,761	16.8	0.5
3–5	1,754,801	16.9	0.5	1,717,175	17	0.8
6–8	1,720,719	16.6	0.6	1,682,635	16.7	0.9
9–11	1,688,790	16.3	0.5	1,647,342	16.3	0.7
12–14	1,703,104	16.4	0.4	1,654,039	16.4	0.8
15–17	1,768,995	17.1	0.2	1,707,842	16.9	0.5
Total	10,374,041	100	0.4	10,104,794	100	0.7

For family characteristics, younger parental age was associated with a higher risk of victimization, as was having a parent born outside Finland. Children living with a single parent and those with three or four siblings had a higher risk of victimization compared with others. The risk of victimization was quite similar across calendar years (results not shown).

### Regression models

Table II presents the results of the regression analyses on the associations of SEP indicators with children’s violent victimization. In the crude model, the odds ratio (OR) of maternal violence was 5.81 (95% **confidence** interval (CI) 5.20–6.50) for mothers with no secondary education, and 2.52 (95% CI 2.27–2.79) for mothers with secondary education compared with tertiary-educated mothers. Mother’s non-employment almost tripled the odds for violence (OR = 2.74, 95% CI 2.52–2.98), while the OR for mothers on social assistance was 5.93 (95% CI 5.44–6.46). The associations with paternal violence were similar, but the relative SEP differences were somewhat smaller. Income had a gradient-like association with violent victimization, with crude ORs ranging between 1.17 and 1.89 among mothers and between 1.36 and 3.46 among fathers, using the highest income group as the reference (Table II).

**Table II.** Crude and adjusted odds ratios of violent victimization by parental SEP variables.

	Mothers				Fathers			
Education	Crude OR	95% CI	Adjusted OR	95% CI	Crude OR	95% CI	Adjusted OR	95% CI
Any tertiary	1.00		1.00		1.00		1.00	
Secondary	2.52	2.27–2.79	1.99	1.79–2.22	1.81	1.68–1.96	1.59	1.47–1.72
No secondary	5.81	5.20–6.50	2.90	2.53–3.31	3.18	2.91–3.47	2.24	2.03–2.46
Social assistance receipt								
No	1.00		1.00		1.00		1.00	
Yes	5.93	5.44–6.46	2.36	2.10–2.64	4.00	3.72–4.31	2.38	2.18–2.59
Non-employed								
No	1.00		1.00		1.00		1	
Yes	2.74	2.52–2.98	1.76	1.60–1.93	2.88	2.67–3.1	1.92	1.76–2.08
Income quintile								
Highest	1.00		1.00		1.00		1.00	
4th	1.17	1.01–1.34	1.14	0.99–1.31	1.36	1.2–1.52	1.29	1.15–1.45
3rd	1.39	1.21–1.60	1.42	1.23–1.63	1.77	1.58–1.99	1.62	1.45–1.82
2nd	1.76	1.54–2.01	1.70	1.49–1.95	2.30	2.06–2.57	1.99	1.78–2.23
Lowest	1.89	1.65–2.16	1.98	1.72–2.28	3.46	3.12–3.84	2.56	2.28–2.86

*Note*: All odds ratios (ORs) of socioeconomic position (SEP) variables were from separate models.

Adjusted OR: adjusted for parent–child living arrangements, parental age, parental country of birth, child’s age and gender, and calendar year.

After covariate adjustment, the socioeconomic differences in children’s violent victimization were attenuated, but the associations remained clear. Maternal basic (OR 2.90, 95% CI 2.53–3.31) and secondary education (OR 1.99, 95% CI 1.79–2.22) remained strong risk factors for violent victimization. Similarly, after covariate adjustment, paternal basic education (OR 2.24, 95% CI 2.03–2.46) and secondary education (OR 1.59, 95% CI 1.47–1.72) increased the odds of victimization when compared with tertiary education. Adjusted ORs of social assistance receipt (OR 2.4, 95% CI 2.10–2.64 for mothers and 2.18–2.59 for fathers) and non-employment (mothers OR 1.8, 95% CI 1.60–1.93; fathers OR 1.9, 95% CI 1.76–2.08) were of similar magnitude among mothers and fathers. Finally, income was associated with victimization in a gradient-like manner, with ORs ranging between 1.14 (95% CI 0.99–1.31) and 1.98 (95% CI 1.72–2.28) among mothers and between 1.29 (95% CI 1.15–1.45) and 2.56 (95% CI 2.28–2.86) among fathers with lower incomes. The results from sensitivity analyses examining the associations of ever experiencing parental violence during the follow-up were very similar to our main results, except for a stronger association between income and maternal violence (Supplementary Table 4).

### Effect modification

**Figure 2.**
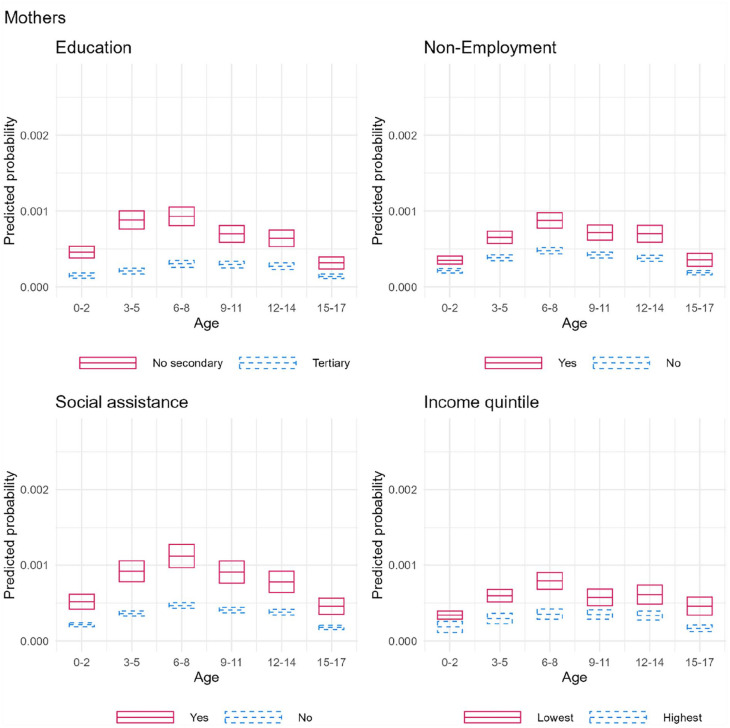
Average predicted probabilities with 95% confidence intervals of child’s violent victimization by interaction between mothers’ SEP and child’s age.

**Figure 3.**
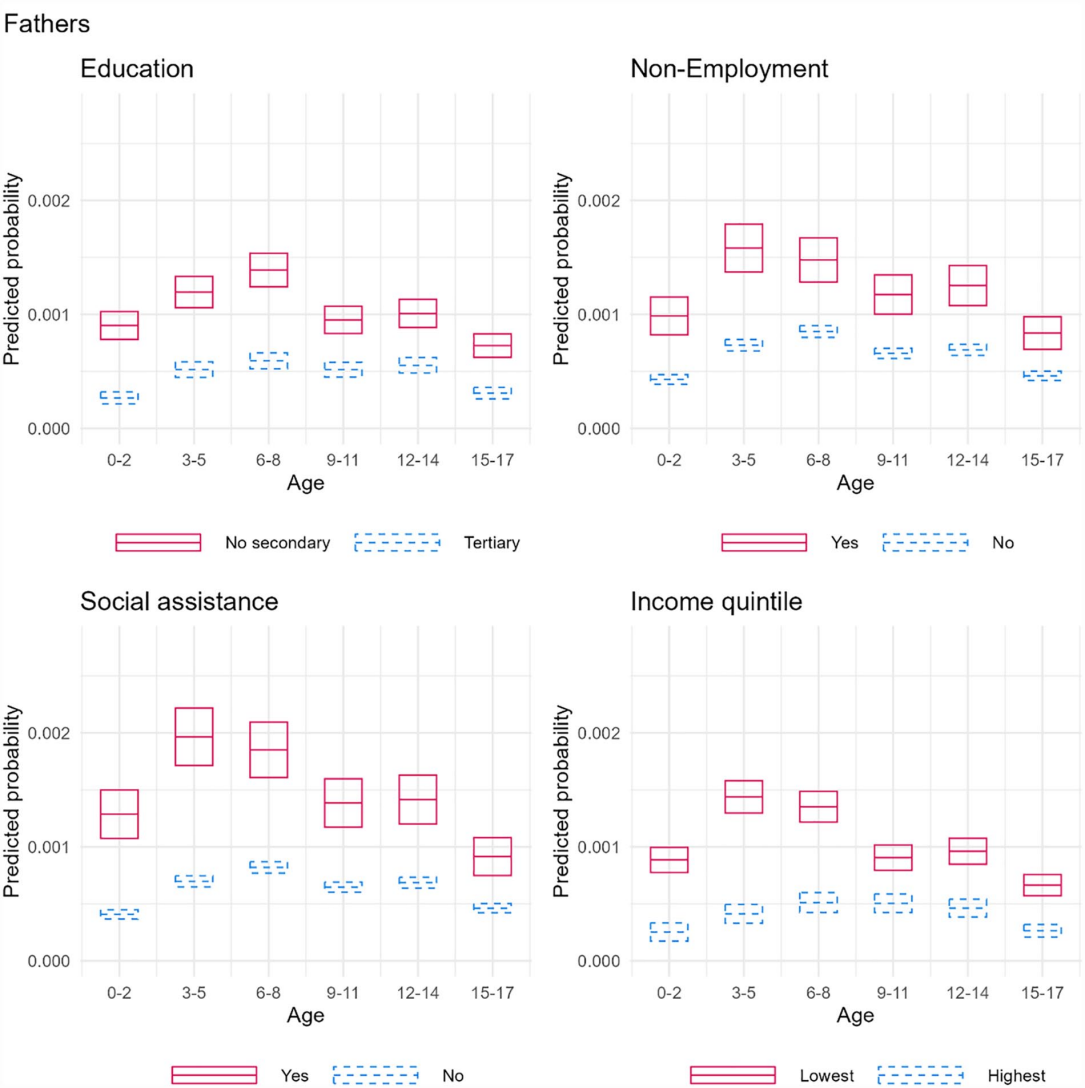
Average predicted probabilities with 95% confidence intervals of child’s violent victimization by interaction between fathers’ SEP and child’s age.

The results from the interaction analyses between parental SEP and child’s age are presented in Figures 2 and 3 as predicted probabilities. For categorical SEP variables, we only present results for the lowest and highest SEP groups. Regarding maternal violence (Figure 2), the predicted probability of victimization was higher among children of mothers with lower SEP at all ages. The overall age patterning of the associations were quite similar with different measures of parental SEP: compared with ages 0–2, the risk of victimization grew until ages 6–8, after which the risk decreased. The absolute difference between the low and high SEP categories increased between ages 0–8 (except for maternal education, where the highest difference was at ages 3–5), after which the risks started to converge. The multiplicative interaction terms from these analyses were not statistically significant (Supplementary Table 1).

The risk of paternal violence was also consistently higher in the lower categories of SEP. However, the socioeconomic differences were somewhat smaller among older children. In contrast to maternal violence, the risk of victimization peaked in the lower SEP categories at ages 3–5, after which the risks started to decrease (except for education), and the largest SEP difference was also observed in this age group. In the higher SEP categories, the increase was slower, and the risks started to decrease only after ages 6–8. In the paternal analyses, many of the multiplicative interaction terms were also statistically significant (Supplementary Table 2).

We also tested for an interaction between child’s gender and parental SEP variables but did not find evidence of effect modification (Supplementary Table 3 and Supplementary Figure 1). However, there were gender differences in the risk of victimization to paternal and maternal violence. Girls were less likely to be a victim of paternal violence than boys, whereas the risk of child’s victimization to maternal violence was similar between boys and girls (Supplementary Figure 1). Finally, we repeated the effect modification analysis by child’s age separately for boys and girls. The age patterns of victimization were different between boys and girls (Supplementary Figures 2–5). Among boys, the highest risk of maternal victimization was at ages 6–8, and of paternal victimization, at ages 3–5 (Supplementary Figure 2). Among girls, the highest risk of victimization occurred at age 12–14 (Supplementary Figure 3). The SEP differences in the risk of maternal violence among boys were biggest at the age of 6–8. In older age groups, the SEP differences were clearly smaller or even null in the oldest age groups (Supplementary Figure 2). Among girls, the SEP differences were highest at age groups 3–5 and 6–8. In older age groups, the differences remained similar, but in the oldest age group the SEP difference in risk was again somewhat smaller, except in relation to income (Supplementary Figure 3). The socioeconomic differences in risk of paternal victimization were relatively similar in all age groups among girls (Supplementary Figure 5). Among boys, the largest difference was in the 3–5 age group. In the older age groups, the SEP differences were smaller but the risk of victimization consistently higher in low SEP groups (Supplementary Figure 4).

## Discussion

We linked total population data to police records on domestic violence to explore and provide new insights into the association between parental SEP and children’s experiences of parental violence by biological parents. According to our analyses, all indicators of low SEP increased the risk of children’s experience of both maternal and paternal physical violence. Children with lower parental SEP were at especially high risk of parental, particularly paternal, violence at ages 3−8, whereas the socioeconomic differences in the risk of violence were somewhat smaller among older children. The overall risk of victimization varied according to the children’s age, with lowest risk for victimization being among the youngest and the oldest children. Lower parental SEP was consistently associated with a higher risk of children’s violent victimization in our analyses, regardless of the SEP measure or children’s age.

Regarding the association between parental SEP and children’s experiences of parental violence, our findings are in line with previous studies showing that low parental SEP is associated with a higher risk of children experiencing violence at home [1,2], but they differ from the most recent findings of Nordic studies based on large survey data [3-6]. This difference could be due to several reasons. If register data mainly capture the most severe forms of violence that result in physical injuries, and the survey items measure violence in a more inclusive way, the two data sources may partly tap into different domains of victimization. Moreover, existing research suggests that socioeconomic differences in adult violent victimization increase with the severity of the violence, according to both survey and register data [15], which could partly explain the differing results. However, detection biases may also contribute to variations in socioeconomic differences between administrative and survey data. Whereas families with low SEP could have more contact with authorities increasing the likelihood of authorities becoming aware of family violence [16], SEP differences may be underestimated in surveys, because of higher rates of nonresponse among lower SEP groups.

We found parental SEP to be an important predictor of children’s victimization to both maternal and paternal violence, and in line with earlier research [3], our analysis underlines the importance of separate assessment of maternal and paternal violence. Finally, the register data enabled us to assess socioeconomic differences in victimization across childhood (ages 0–17). New to the existing literature, there is no direct point of reference in previous research for our findings on the risks of victimization among children with lower parental SEP peaking at ages 3−8. The overall association between children’s age and the risk of victimization was also somewhat different in our study compared with previous Finnish survey data suggesting that the risks peak at ages 3−6 and 14−15 among both genders [13], but this could possibly be explained by the greater accuracy of administrative data.

### Strengths and limitations

The key strengths of our analysis are the total population coverage of longitudinal register data, reliable measurement of parental SEP via multiple measures for both mothers and fathers, and the consistent definition of parental violence following legal standards. These kinds of data are available in the Nordic countries but unique in a larger international context. As suggested in existing literature [8,11], Nordic countries with register data could play a prominent role in establishing a sustainable infrastructure of robust and comprehensive child maltreatment data that can be routinely used in an ethical way to obtain information on violence against children. The main shortcoming of administrative data lies in a potentially biased reporting of violent incidents to authorities, and some detection biases may be related to administrative data, as described above.

In addition to the availability of the unique data sources, the Nordic welfare state is a specific context in terms of social equality. In Finland, income equality and the educational level of the population are internationally high, and the school system and health and social welfare services are free-of-charge. It is relatively hard to estimate whether the findings on SEP differences presented here would be replicated in other contexts, but previous studies do suggest that SEP differences in child victimization occur in other types of societies as well [1,2], although these differences might be of a different magnitude.

## Conclusions and future research directions

In this study, we focused on parental SEP, but register data could also be used to study the role of other parental- and family-level risk markers of children’s violent victimization, such as parental mental health problems, parental criminal behavior, and parental victimization [1,2]. At its best, longitudinal register data enable the building of models that provide further insights into the causal processes leading to child victimization.

Our findings about the association between parental SEP and parental violence should be considered in planning and providing services to families with children. The importance of socioeconomic resources in parental practices should be acknowledged in child and maternity clinics, schools, and in child protective services when evaluating the risk for abusive parental practices. Supportive measures for low SEP parents might be in order to prevent parental violence or to intervene in ongoing abuse. These measures could be implemented across different institutions, such as health and social care services and the school system, and could consist of support in parenting practices and referrals to treatment when in need, among others.

However, in this study, we did not assess the causal nature of the association between SEP and parental violence toward their children. For effective prevention of child victimization and design of suitable interventions, further research on the etiology of child victimization and the mechanisms underlying the observed SEP differences is needed.

## Supplemental Material

sj-docx-1-sjp-10.1177_14034948231180670 – Supplemental material for Socioeconomic differences in children’s victimization to maternal and paternal violence: a register-based study Supplemental material, sj-docx-1-sjp-10.1177_14034948231180670 for Socioeconomic differences in children’s victimization to maternal and paternal violence: a register-based study by NOORA ELLONEN, JOONAS PITKÄNEN, MIKKO AALTONEN, HANNA REMES and PEKKA MARTIKAINEN in Scandinavian Journal of Public Health
