# Supplementary material for: Socioeconomic differences in children’s victimization to maternal and paternal violence: a register-based study
Source: Scand J Public Health. 2023 Aug 17;52(7):800–9. doi: 10.1177/14034948231180670 (PMC11490066; doi:10.1177/14034948231180670)
Supplement: sj-docx-1-sjp-10.1177_14034948231180670 – Supplemental material for Socioeconomic differences in children’s victimization to maternal and paternal violence: a register-based study [file sj-docx-1-sjp-10.1177_14034948231180670.docx]

| Supplementary Table 1: Odds Ratios and 95% Cis from interaction analyses between child’s age and parental SEP. Mother-child data | | | | | | | | | | |
| --- | --- | --- | --- | --- | --- | --- | --- | --- | --- | --- |
|  | Total | | | Boys | | | Girls | | | |
|  | OR | 95% CI | | OR | 95% CI | | OR | 95% CI | |  |
| **Age X Maternal education** |  |  |  |  |  |  |  |  |  |  |
| Age group (ref. 0-2-year-olds) |  |  |  |  |  |  |  |  |  |  |
| 3-5 | 1.41 | 1.09 | 1.83 | 1.33 | 0.94 | 1.88 | 1.51 | 1.02 | 2.22 |  |
| 6-8 | 2.05 | 1.59 | 2.65 | 2.22 | 1.61 | 3.07 | 1.79 | 1.21 | 2.64 |  |
| 9-11 | 1.99 | 1.52 | 2.61 | 1.91 | 1.35 | 2.71 | 2.07 | 1.40 | 3.06 |  |
| 12-14 | 1.84 | 1.38 | 2.44 | 1.03 | 0.70 | 1.53 | 3.01 | 2.01 | 4.50 |  |
| 15-17 | 0.94 | 0.67 | 1.30 | 0.40 | 0.23 | 0.69 | 1.73 | 1.12 | 2.67 |  |
| Maternal education (ref. Any tertiary) |  |  |  |  |  |  |  |  |  |  |
| Secondary | 2.13 | 1.62 | 2.81 | 1.92 | 1.36 | 2.71 | 2.40 | 1.61 | 3.57 |  |
| No secondary | 3.10 | 2.32 | 4.15 | 3.09 | 2.15 | 4.46 | 3.11 | 2.04 | 4.72 |  |
| Age group x maternal education |  |  |  |  |  |  |  |  |  |  |
| 3-5 X No secondary | 1.36 | 1.00 | 1.86 | 1.37 | 0.91 | 2.08 | 1.35 | 0.85 | 2.13 |  |
| 3-5 X Secondary | 1.17 | 0.86 | 1.59 | 1.35 | 0.90 | 2.03 | 0.99 | 0.63 | 1.56 |  |
| 6-8 X No secondary | 0.99 | 0.72 | 1.35 | 0.91 | 0.61 | 1.36 | 1.12 | 0.70 | 1.81 |  |
| 6-8 X Secondary | 1.04 | 0.77 | 1.40 | 1.11 | 0.75 | 1.63 | 0.98 | 0.62 | 1.54 |  |
| 9-11 X No secondary | 0.76 | 0.55 | 1.07 | 0.69 | 0.44 | 1.08 | 0.86 | 0.53 | 1.40 |  |
| 9-11 X Secondary | 0.86 | 0.63 | 1.19 | 0.85 | 0.56 | 1.28 | 0.89 | 0.56 | 1.40 |  |
| 12-14 X No secondary | 0.76 | 0.53 | 1.08 | 0.66 | 0.39 | 1.12 | 0.81 | 0.50 | 1.31 |  |
| 12-14 X Secondary | 0.80 | 0.57 | 1.11 | 0.78 | 0.49 | 1.26 | 0.75 | 0.48 | 1.18 |  |
| 15-17 X No secondary | 0.73 | 0.48 | 1.13 | 0.67 | 0.31 | 1.45 | 0.76 | 0.44 | 1.30 |  |
| 15-17 X Secondary | 0.73 | 0.50 | 1.08 | 0.83 | 0.43 | 1.60 | 0.65 | 0.39 | 1.07 |  |
| **Age X maternal non-employment** |  |  |  |  |  |  |  |  |  |  |
| Age group (ref. 0-2-year-olds) |  |  |  |  |  |  |  |  |  |  |
| 3-5 | 1.80 | 1.54 | 2.09 | 1.78 | 1.46 | 2.18 | 1.80 | 1.43 | 2.27 |  |
| 6-8 | 2.23 | 1.91 | 2.60 | 2.33 | 1.91 | 2.85 | 2.05 | 1.62 | 2.59 |  |
| 9-11 | 1.97 | 1.66 | 2.33 | 1.82 | 1.47 | 2.26 | 2.13 | 1.66 | 2.74 |  |
| 12-14 | 1.77 | 1.48 | 2.11 | 0.91 | 0.70 | 1.18 | 3.00 | 2.33 | 3.85 |  |
| 15-17 | 0.88 | 0.71 | 1.09 | 0.39 | 0.28 | 0.55 | 1.58 | 1.19 | 2.11 |  |
| Maternal non-employment (ref. No) |  |  |  |  |  |  |  |  |  |  |
| Yes | 1.65 | 1.36 | 2.00 | 1.51 | 1.18 | 1.95 | 1.81 | 1.38 | 2.37 |  |
| Age group X maternal non-employment | |  |  |  |  |  |  |  |  |  |
| 3-5 X Yes | 1.04 | 0.84 | 1.28 | 1.05 | 0.78 | 1.41 | 1.03 | 0.75 | 1.41 |  |
| 6-8 X Yes | 1.12 | 0.89 | 1.40 | 1.19 | 0.89 | 1.59 | 1.05 | 0.75 | 1.47 |  |
| 9-11 X Yes | 1.03 | 0.81 | 1.31 | 0.94 | 0.67 | 1.31 | 1.13 | 0.80 | 1.60 |  |
| 12-14 Yes | 1.13 | 0.87 | 1.46 | 1.19 | 0.80 | 1.78 | 1.05 | 0.74 | 1.47 |  |
| 15-17 X Yes | 1.16 | 0.83 | 1.61 | 0.93 | 0.50 | 1.74 | 1.17 | 0.78 | 1.75 |  |
| **Age X maternal social asistance** |  |  |  |  |  |  |  |  |  |  |
| Age group (ref. 0-2-year-olds) |  |  |  |  |  |  |  |  |  |  |
| 3-5 | 1.69 | 1.48 | 1.93 | 1.75 | 1.45 | 2.10 | 1.62 | 1.33 | 1.97 |  |
| 6-8 | 2.18 | 1.90 | 2.50 | 2.44 | 2.03 | 2.94 | 1.84 | 1.49 | 2.27 |  |
| 9-11 | 1.90 | 1.63 | 2.22 | 1.73 | 1.41 | 2.13 | 2.10 | 1.68 | 2.62 |  |
| 12-14 | 1.77 | 1.50 | 2.09 | 0.93 | 0.73 | 1.19 | 2.86 | 2.29 | 3.59 |  |
| 15-17 | 0.84 | 0.68 | 1.03 | 0.33 | 0.23 | 0.46 | 1.51 | 1.16 | 1.96 |  |
| Maternal social assistance (ref. No) |  |  |  |  |  |  |  |  |  |  |
| Yes | 2.41 | 1.93 | 3.01 | 2.52 | 1.89 | 3.35 | 2.28 | 1.67 | 3.10 |  |
| Age group X maternal social assistance | |  |  |  |  |  |  |  |  |  |
| 3-5 X Yes | 1.05 | 0.84 | 1.32 | 0.98 | 0.72 | 1.32 | 1.15 | 0.83 | 1.61 |  |
| 6-8 X Yes | 1.00 | 0.79 | 1.27 | 0.90 | 0.66 | 1.23 | 1.15 | 0.81 | 1.64 |  |
| 9-11 X Yes | 0.93 | 0.72 | 1.20 | 0.97 | 0.69 | 1.37 | 0.88 | 0.60 | 1.28 |  |
| 12-14 Yes | 0.85 | 0.64 | 1.13 | 0.94 | 0.61 | 1.44 | 0.85 | 0.59 | 1.23 |  |
| 15-17 X Yes | 1.06 | 0.76 | 1.48 | 1.54 | 0.88 | 2.68 | 0.97 | 0.63 | 1.48 |  |
| **Age X maternal income** |  |  |  |  |  |  |  |  |  |  |
| Age group (ref. 0-2-year-olds) |  |  |  |  |  |  |  |  |  |  |
| 3-5 | 1.59 | 1.04 | 2.44 | 1.29 | 0.75 | 2.20 | 2.19 | 1.05 | 4.55 |  |
| 6-8 | 1.90 | 1.26 | 2.86 | 1.51 | 0.92 | 2.48 | 2.66 | 1.31 | 5.41 |  |
| 9-11 | 1.87 | 1.22 | 2.88 | 1.63 | 0.98 | 2.71 | 2.32 | 1.12 | 4.80 |  |
| 12-14 | 1.80 | 1.17 | 2.77 | 0.89 | 0.52 | 1.54 | 3.71 | 1.83 | 7.52 |  |
| 15-17 | 0.90 | 0.56 | 1.45 | 0.29 | 0.14 | 0.62 | 2.18 | 1.04 | 4.58 |  |
| Maternal income quintile (ref. Highest) |  |  |  |  |  |  |  |  |  |  |
| 2nd | 1.42 | 0.91 | 2.20 | 1.13 | 0.67 | 1.90 | 1.98 | 0.95 | 4.10 |  |
| 3rd | 1.38 | 0.86 | 2.20 | 1.35 | 0.79 | 2.33 | 1.45 | 0.67 | 3.14 |  |
| 4th | 1.17 | 0.70 | 1.97 | 0.92 | 0.49 | 1.74 | 1.69 | 0.75 | 3.78 |  |
| Lowest | 1.84 | 1.20 | 2.82 | 1.48 | 0.90 | 2.43 | 2.55 | 1.25 | 5.18 |  |
| Age group X maternal income |  |  |  |  |  |  |  |  |  |  |
| 3-5 X 4th quintile | 0.99 | 0.57 | 1.69 | 1.32 | 0.65 | 2.70 | 0.65 | 0.27 | 1.57 |  |
| 3-5 X 3rd quintile | 1.04 | 0.63 | 1.71 | 1.09 | 0.58 | 2.05 | 0.95 | 0.41 | 2.20 |  |
| 3-5 X 2nd quintile | 1.34 | 0.83 | 2.15 | 1.85 | 1.01 | 3.40 | 0.84 | 0.38 | 1.85 |  |
| 3-5 X Lowest quintile | 1.10 | 0.70 | 1.74 | 1.36 | 0.76 | 2.45 | 0.79 | 0.37 | 1.71 |  |
| 6-8 X 4th quintile | 0.97 | 0.57 | 1.65 | 1.45 | 0.73 | 2.88 | 0.54 | 0.23 | 1.28 |  |
| 6-8 X 3rd quintile | 1.31 | 0.80 | 2.13 | 1.63 | 0.90 | 2.95 | 0.92 | 0.40 | 2.09 |  |
| 6-8 X 2nd quintile | 1.38 | 0.87 | 2.19 | 1.88 | 1.07 | 3.32 | 0.88 | 0.41 | 1.92 |  |
| 6-8 X Lowest quintile | 1.22 | 0.78 | 1.91 | 1.79 | 1.03 | 3.12 | 0.70 | 0.33 | 1.50 |  |
| 9-11 X 4th quintile | 1.03 | 0.59 | 1.79 | 1.16 | 0.58 | 2.33 | 0.83 | 0.35 | 2.01 |  |
| 9-11 X 3rd quintile | 1.01 | 0.60 | 1.68 | 0.95 | 0.51 | 1.76 | 1.06 | 0.46 | 2.45 |  |
| 9-11 X 2nd quintile | 1.21 | 0.75 | 1.97 | 1.26 | 0.69 | 2.30 | 1.08 | 0.49 | 2.39 |  |
| 9-11 X Lowest quintile | 0.90 | 0.55 | 1.46 | 0.84 | 0.46 | 1.54 | 0.88 | 0.40 | 1.93 |  |
| 12-14 X 4th quintile | 0.91 | 0.52 | 1.60 | 0.90 | 0.42 | 1.95 | 0.71 | 0.30 | 1.65 |  |
| 12-14 X 3rd quintile | 0.86 | 0.51 | 1.45 | 0.92 | 0.47 | 1.82 | 0.79 | 0.35 | 1.79 |  |
| 12-14 X 2nd quintile | 1.19 | 0.73 | 1.94 | 1.26 | 0.64 | 2.46 | 0.91 | 0.42 | 1.98 |  |
| 12-14 X Lowest quintile | 0.99 | 0.61 | 1.62 | 0.99 | 0.50 | 1.96 | 0.79 | 0.37 | 1.69 |  |
| 15-17 X 4th quintile | 0.95 | 0.51 | 1.76 | 1.67 | 0.64 | 4.35 | 0.59 | 0.24 | 1.45 |  |
| 15-17 X 3rd quintile | 0.79 | 0.44 | 1.43 | 1.10 | 0.43 | 2.77 | 0.67 | 0.28 | 1.61 |  |
| 15-17 X 2nd quintile | 0.77 | 0.43 | 1.39 | 1.17 | 0.44 | 3.06 | 0.52 | 0.22 | 1.22 |  |
| 15-17 X Lowest quintile | 1.50 | 0.86 | 2.60 | 1.31 | 0.50 | 3.40 | 1.19 | 0.53 | 2.65 |  |

| Supplementary Table 2: Odds Ratios and 95% Cis from interaction analyses between child’s age and parental SEP. Father-child data | | | | | | | | | | |
| --- | --- | --- | --- | --- | --- | --- | --- | --- | --- | --- |
|  | Total | | | Boys | | | Girls | | | |
|  | OR | 95% CI | | OR | 95% CI | | OR | 95% CI | |  |
| **Age X paternal education** |  |  |  |  |  |  |  |  |  |  |
| Age group (ref. 0-2-year-olds) |  |  |  |  |  |  |  |  |  |  |
| 3-5 | 1.93 | 1.57 | 2.37 | 1.85 | 1.41 | 2.43 | 2.08 | 1.48 | 2.91 |  |
| 6-8 | 2.22 | 1.78 | 2.76 | 2.18 | 1.66 | 2.87 | 2.26 | 1.60 | 3.21 |  |
| 9-11 | 1.93 | 1.53 | 2.43 | 1.64 | 1.23 | 2.19 | 2.50 | 1.74 | 3.59 |  |
| 12-14 | 2.07 | 1.64 | 2.61 | 1.42 | 1.06 | 1.92 | 3.40 | 2.38 | 4.86 |  |
| 15-17 | 1.16 | 0.89 | 1.50 | 0.81 | 0.57 | 1.14 | 1.88 | 1.27 | 2.76 |  |
| Paternal education (ref. Any tertiary) |  |  |  |  |  |  |  |  |  |  |
| Secondary | 1.82 | 1.46 | 2.28 | 1.65 | 1.25 | 2.18 | 2.14 | 1.51 | 3.04 |  |
| No secondary | 3.38 | 2.68 | 4.27 | 3.35 | 2.51 | 4.47 | 3.46 | 2.39 | 5.02 |  |
| Age group x paternal education |  |  |  |  |  |  |  |  |  |  |
| 3-5 X No secondary | 0.69 | 0.53 | 0.89 | 0.71 | 0.51 | 0.99 | 0.64 | 0.42 | 0.98 |  |
| 3-5 X Secondary | 0.98 | 0.77 | 1.24 | 1.05 | 0.77 | 1.44 | 0.87 | 0.58 | 1.28 |  |
| 6-8 X No secondary | 0.69 | 0.53 | 0.91 | 0.70 | 0.50 | 0.98 | 0.68 | 0.45 | 1.05 |  |
| 6-8 X Secondary | 0.90 | 0.70 | 1.16 | 0.99 | 0.72 | 1.35 | 0.77 | 0.52 | 1.16 |  |
| 9-11 X No secondary | 0.55 | 0.41 | 0.73 | 0.62 | 0.44 | 0.88 | 0.44 | 0.28 | 0.70 |  |
| 9-11 X Secondary | 0.80 | 0.61 | 1.05 | 0.96 | 0.69 | 1.34 | 0.60 | 0.40 | 0.92 |  |
| 12-14 X No secondary | 0.54 | 0.41 | 0.72 | 0.55 | 0.38 | 0.79 | 0.52 | 0.34 | 0.80 |  |
| 12-14 X Secondary | 0.75 | 0.58 | 0.98 | 0.88 | 0.62 | 1.24 | 0.61 | 0.41 | 0.92 |  |
| 15-17 X No secondary | 0.69 | 0.51 | 0.94 | 0.68 | 0.45 | 1.02 | 0.70 | 0.44 | 1.10 |  |
| 15-17 X Secondary | 0.92 | 0.69 | 1.22 | 1.04 | 0.71 | 1.52 | 0.77 | 0.50 | 1.18 |  |
| **Age X paternal non-employment** |  |  |  |  |  |  |  |  |  |  |
| Age group (ref. 0-2-year-olds) |  |  |  |  |  |  |  |  |  |  |
| 3-5 | 1.70 | 1.54 | 1.89 | 1.72 | 1.51 | 1.95 | 1.67 | 1.42 | 1.98 |  |
| 6-8 | 1.99 | 1.78 | 2.21 | 2.06 | 1.80 | 2.35 | 1.84 | 1.54 | 2.20 |  |
| 9-11 | 1.54 | 1.36 | 1.73 | 1.45 | 1.25 | 1.68 | 1.68 | 1.39 | 2.04 |  |
| 12-14 | 1.61 | 1.42 | 1.83 | 1.19 | 1.02 | 1.40 | 2.38 | 1.96 | 2.89 |  |
| 15-17 | 1.08 | 0.93 | 1.24 | 0.81 | 0.67 | 0.97 | 1.58 | 1.28 | 1.95 |  |
| paternal non-employment (ref. No) |  |  |  |  |  |  |  |  |  |  |
| Yes | 2.31 | 1.91 | 2.78 | 2.17 | 1.72 | 2.74 | 2.54 | 1.92 | 3.35 |  |
| Age group X paternal non-employment |  |  |  |  |  |  |  |  |  |  |
| 3-5 X Yes | 0.94 | 0.76 | 1.17 | 0.91 | 0.69 | 1.20 | 1.00 | 0.72 | 1.39 |  |
| 6-8 X Yes | 0.75 | 0.60 | 0.94 | 0.72 | 0.55 | 0.96 | 0.82 | 0.58 | 1.16 |  |
| 9-11 X Yes | 0.78 | 0.61 | 0.98 | 0.83 | 0.62 | 1.12 | 0.69 | 0.48 | 1.00 |  |
| 12-14 Yes | 0.79 | 0.62 | 1.00 | 0.78 | 0.58 | 1.07 | 0.77 | 0.55 | 1.08 |  |
| 15-17 X Yes | 0.79 | 0.61 | 1.02 | 0.70 | 0.49 | 1.00 | 0.84 | 0.59 | 1.21 |  |
| **Age X paternal social asistance** |  |  |  |  |  |  |  |  |  |  |
| Age group (ref. 0-2-year-olds) |  |  |  |  |  |  |  |  |  |  |
| 3-5 | 1.71 | 1.54 | 1.90 | 1.65 | 1.45 | 1.88 | 1.81 | 1.53 | 2.16 |  |
| 6-8 | 2.01 | 1.80 | 2.25 | 2.02 | 1.77 | 2.31 | 1.97 | 1.64 | 2.36 |  |
| 9-11 | 1.59 | 1.41 | 1.79 | 1.48 | 1.28 | 1.72 | 1.77 | 1.45 | 2.15 |  |
| 12-14 | 1.68 | 1.48 | 1.91 | 1.21 | 1.03 | 1.41 | 2.63 | 2.16 | 3.20 |  |
| 15-17 | 1.13 | 0.98 | 1.31 | 0.81 | 0.68 | 0.98 | 1.77 | 1.43 | 2.19 |  |
| paternal social assistance (ref. No) |  |  |  |  |  |  |  |  |  |  |
| Yes | 3.16 | 2.63 | 3.80 | 2.65 | 2.11 | 3.33 | 4.08 | 3.09 | 5.39 |  |
| Age group X paternal social assistance | |  |  |  |  |  |  |  |  |  |
| 3-5 X Yes | 0.89 | 0.73 | 1.09 | 1.05 | 0.81 | 1.35 | 0.71 | 0.52 | 0.98 |  |
| 6-8 X Yes | 0.72 | 0.58 | 0.89 | 0.79 | 0.60 | 1.03 | 0.64 | 0.46 | 0.90 |  |
| 9-11 X Yes | 0.68 | 0.54 | 0.86 | 0.74 | 0.55 | 1.00 | 0.60 | 0.42 | 0.86 |  |
| 12-14 Yes | 0.65 | 0.52 | 0.83 | 0.76 | 0.56 | 1.05 | 0.52 | 0.37 | 0.74 |  |
| 15-17 X Yes | 0.63 | 0.48 | 0.81 | 0.67 | 0.47 | 0.96 | 0.54 | 0.38 | 0.79 |  |
| **Age X paternal income** |  |  |  |  |  |  |  |  |  |  |
| Age group (ref. 0-2-year-olds) |  |  |  |  |  |  |  |  |  |  |
| 3-5 | 1.63 | 1.16 | 2.29 | 1.53 | 0.99 | 2.37 | 1.84 | 0.99 | 3.42 |  |
| 6-8 | 2.02 | 1.42 | 2.88 | 2.00 | 1.31 | 3.04 | 2.05 | 1.10 | 3.83 |  |
| 9-11 | 1.99 | 1.41 | 2.83 | 1.73 | 1.13 | 2.64 | 2.58 | 1.39 | 4.79 |  |
| 12-14 | 1.83 | 1.28 | 2.62 | 1.29 | 0.83 | 2.00 | 3.04 | 1.62 | 5.70 |  |
| 15-17 | 1.04 | 0.71 | 1.53 | 0.63 | 0.38 | 1.03 | 1.99 | 1.04 | 3.80 |  |
| paternal income quintile (ref. Highest) |  |  |  |  |  |  |  |  |  |  |
| 2nd | 1.13 | 0.76 | 1.68 | 1.10 | 0.69 | 1.75 | 1.21 | 0.60 | 2.43 |  |
| 3rd | 1.59 | 1.10 | 2.30 | 1.55 | 1.00 | 2.40 | 1.69 | 0.88 | 3.26 |  |
| 4th | 1.98 | 1.39 | 2.82 | 1.74 | 1.13 | 2.67 | 2.51 | 1.34 | 4.67 |  |
| Lowest | 3.50 | 2.50 | 4.91 | 3.09 | 2.07 | 4.63 | 4.39 | 2.39 | 8.05 |  |
| Age group X paternal income |  |  |  |  |  |  |  |  |  |  |
| 3-5 X 4th quintile | 1.05 | 0.68 | 1.62 | 1.14 | 0.66 | 1.99 | 0.87 | 0.40 | 1.88 |  |
| 3-5 X 3rd quintile | 1.07 | 0.71 | 1.60 | 1.07 | 0.64 | 1.79 | 1.05 | 0.51 | 2.16 |  |
| 3-5 X 2nd quintile | 1.19 | 0.81 | 1.75 | 1.29 | 0.79 | 2.12 | 1.02 | 0.52 | 2.01 |  |
| 3-5 X Lowest quintile | 1.00 | 0.69 | 1.44 | 1.07 | 0.67 | 1.71 | 0.87 | 0.45 | 1.68 |  |
| 6-8 X 4th quintile | 1.11 | 0.72 | 1.73 | 1.13 | 0.67 | 1.91 | 1.07 | 0.50 | 2.31 |  |
| 6-8 X 3rd quintile | 1.09 | 0.72 | 1.64 | 1.02 | 0.62 | 1.68 | 1.21 | 0.59 | 2.48 |  |
| 6-8 X 2nd quintile | 1.13 | 0.76 | 1.68 | 1.30 | 0.80 | 2.10 | 0.87 | 0.44 | 1.74 |  |
| 6-8 X Lowest quintile | 0.76 | 0.52 | 1.11 | 0.77 | 0.49 | 1.22 | 0.73 | 0.38 | 1.42 |  |
| 9-11 X 4th quintile | 0.99 | 0.64 | 1.55 | 1.06 | 0.62 | 1.81 | 0.88 | 0.41 | 1.89 |  |
| 9-11 X 3rd quintile | 0.92 | 0.61 | 1.39 | 0.97 | 0.59 | 1.60 | 0.83 | 0.40 | 1.71 |  |
| 9-11 X 2nd quintile | 0.81 | 0.55 | 1.21 | 0.89 | 0.54 | 1.46 | 0.68 | 0.34 | 1.35 |  |
| 9-11 X Lowest quintile | 0.51 | 0.35 | 0.75 | 0.60 | 0.38 | 0.96 | 0.38 | 0.20 | 0.75 |  |
| 12-14 X 4th quintile | 1.32 | 0.84 | 2.06 | 1.28 | 0.74 | 2.21 | 1.31 | 0.61 | 2.79 |  |
| 12-14 X 3rd quintile | 0.96 | 0.63 | 1.47 | 1.02 | 0.60 | 1.71 | 0.88 | 0.43 | 1.82 |  |
| 12-14 X 2nd quintile | 0.95 | 0.64 | 1.43 | 1.07 | 0.64 | 1.78 | 0.77 | 0.39 | 1.54 |  |
| 12-14 X Lowest quintile | 0.59 | 0.40 | 0.88 | 0.60 | 0.37 | 0.98 | 0.53 | 0.27 | 1.04 |  |
| 15-17 X 4th quintile | 1.54 | 0.96 | 2.46 | 1.89 | 1.03 | 3.46 | 1.24 | 0.57 | 2.71 |  |
| 15-17 X 3rd quintile | 1.27 | 0.81 | 1.99 | 1.49 | 0.83 | 2.66 | 1.08 | 0.51 | 2.26 |  |
| 15-17 X 2nd quintile | 1.06 | 0.68 | 1.64 | 1.57 | 0.89 | 2.77 | 0.66 | 0.32 | 1.35 |  |
| 15-17 X Lowest quintile | 0.72 | 0.47 | 1.09 | 0.79 | 0.46 | 1.37 | 0.60 | 0.30 | 1.18 |  |

| Supplementary Table 3: Odds Ratios and 95% Cis from intearction analyses between child gender and SEP variables. | | | | | | |
| --- | --- | --- | --- | --- | --- | --- |
|  | Maternal violence | | | Paternal violence | | |
|  | OR | 95% CI | | OR | 95% CI | |
| **Gender X Parental education** |  |  |  |  |  |  |
| Parental education (ref. Any tertiary) |  |  |  |  |  |  |
| No secondary | 2.88 | 2.45 | 3.39 | 2.31 | 2.06 | 2.59 |
| Secondary | 1.94 | 1.70 | 2.22 | 1.62 | 1.47 | 1.79 |
| Child's gender (ref. Boy) |  |  |  |  |  |  |
| Girl | 1.01 | 0.88 | 1.16 | 0.76 | 0.68 | 0.85 |
| Gender X Parental education |  |  |  |  |  |  |
| No secondary X girl | 1.01 | 0.84 | 1.21 | 0.92 | 0.80 | 1.07 |
| Secondary X girl | 1.05 | 0.89 | 1.24 | 0.95 | 0.84 | 1.09 |
| **Gender X Non-employment** |  |  |  |  |  |  |
| Parental non-employment (ref. employed) | |  |  |  |  |  |
| Yes | 1.73 | 1.54 | 1.94 | 1.80 | 1.63 | 1.99 |
| Child's gender (ref. Boy) |  |  |  |  |  |  |
| Girl | 1.03 | 0.95 | 1.11 | 0.71 | 0.67 | 0.75 |
| Gender X Parental non-employment |  |  |  |  |  |  |
| Yes X girl | 1.04 | 0.91 | 1.19 | 1.15 | 1.02 | 1.31 |
| **Gender X Social assistance** |  |  |  |  |  |  |
| Parental social assistance (ref. no receipt) | |  |  |  |  |  |
| Yes | 2.52 | 2.20 | 2.88 | 2.28 | 2.06 | 2.52 |
| Child's gender (ref. Boy) |  |  |  |  |  |  |
| Girl | 1.08 | 1.00 | 1.17 | 0.71 | 0.67 | 0.75 |
| Gender X Parental social asistance |  |  |  |  |  |  |
| Yes X girl | 0.87 | 0.76 | 1.00 | 1.11 | 0.98 | 1.25 |
| **Gender X parental income** |  |  |  |  |  |  |
| Income quintile (ref. Highest) |  |  |  |  |  |  |
| 4th quintile | 1.14 | 0.95 | 1.38 | 1.31 | 1.13 | 1.51 |
| 3rd quintile | 1.60 | 1.34 | 1.92 | 1.64 | 1.43 | 1.89 |
| 2nd quintile | 1.79 | 1.50 | 2.13 | 2.04 | 1.78 | 2.34 |
| Lowest quintile | 1.97 | 1.64 | 2.37 | 2.51 | 2.19 | 2.87 |
| Child's gender (ref. Boy) |  |  |  |  |  |  |
| Girl | 1.12 | 0.94 | 1.32 | 0.74 | 0.63 | 0.86 |
| Gender X parental income |  |  |  |  |  |  |
| 4th quintile X girl | 0.99 | 0.79 | 1.24 | 0.97 | 0.79 | 1.18 |
| 3rd quintile X girl | 0.78 | 0.62 | 0.97 | 0.97 | 0.80 | 1.18 |
| 2nd quintile X girl | 0.91 | 0.74 | 1.12 | 0.94 | 0.78 | 1.13 |
| Lowest quintile X girl | 1.01 | 0.81 | 1.24 | 1.05 | 0.88 | 1.25 |

| STable  4: Crude and Adjusted Odds Ratios of ever experiencing violent victimization by parental SEP variables | | | | | | | | |
| --- | --- | --- | --- | --- | --- | --- | --- | --- |
|  | Mothers | | | | Fathers | | | |
| **Education** | Crude OR | 95% CI | Adjusted OR | 95% CI | Crude OR | 95% CI | Adjusted OR | 95% CI |
| Any Tertiary | 1 |  | 1 |  | 1 |  | 1 |  |
| Secondary | 2.42 | 2.18,2.69 | 2.09 | 1.88,2.33 | 1.75 | 1.62,1.9 | 1.62 | 1.5,1.76 |
| No secondary | 5.92 | 5.31,6.61 | 3.17 | 2.78,3.63 | 3.17 | 2.91,3.46 | 2.46 | 2.23,2.7 |
| **Social assistance receipt** |  |  |  |  |  |  |  |  |
| No | 1 |  | 1 |  | 1 |  | 1 |  |
| Yes | 4.98 | 4.56,5.45 | 2.31 | 2.05,2.61 | 3.46 | 3.2,3.74 | 2.34 | 2.14,2.56 |
| **Non-employed** |  |  |  |  |  |  |  |  |
| No | 1 |  | 1 |  | 1 |  | 1 |  |
| Yes | 2.64 | 2.44,2.86 | 1.63 | 1.49,1.78 | 2.51 | 2.32,2.72 | 1.88 | 1.72,2.06 |
| **Income quintile** |  |  |  |  |  |  |  |  |
| Highest | 1 |  | 1 |  | 1 |  | 1 |  |
| 4th | 1.27 | 1.07,1.51 | 1.16 | 0.98,1.37 | 1.3 | 1.16,1.47 | 1.26 | 1.11,1.42 |
| 3rd | 1.54 | 1.31,1.81 | 1.32 | 1.12,1.56 | 1.58 | 1.41,1.78 | 1.48 | 1.32,1.66 |
| 2nd | 2.31 | 1.98,2.68 | 1.65 | 1.41,1.92 | 2.09 | 1.87,2.33 | 1.86 | 1.66,2.08 |
| Lowest | 3.16 | 2.73,3.66 | 2 | 1.72,2.33 | 3.33 | 3,3.7 | 2.62 | 2.35,2.93 |
| All ORs of SES variables from separate models | | | | | | | | |
| Adjusted Ors adjusted for parent-child living arrangements, parental age, parental country of birth, child's gender and child's year of birth. Control variables derived from first observation in the data between age 0-17  SEP measured at first observation in the data | | | | | | | | |


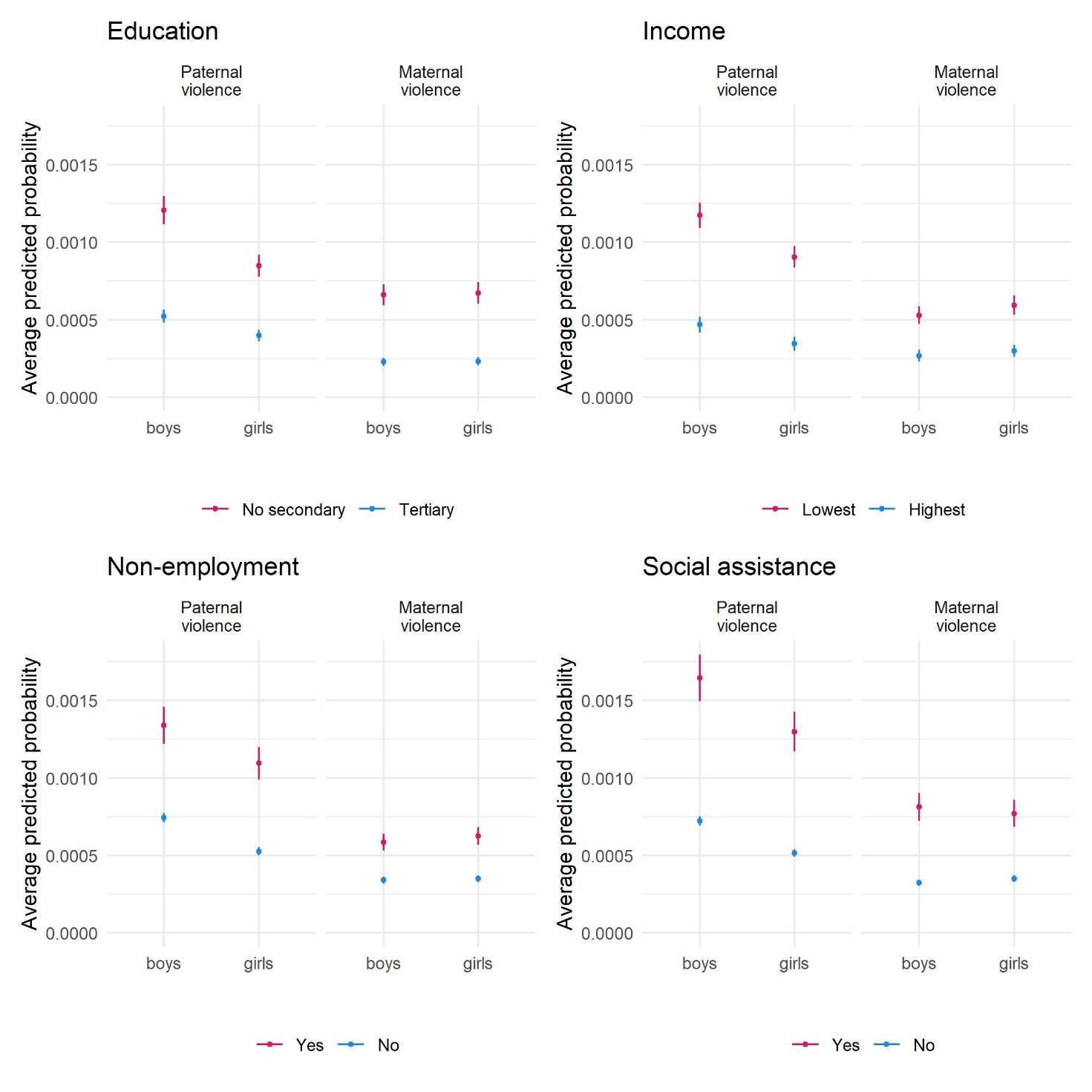


Figure 1 Predicted probabilities from interaction analyses between parental SEP and child’s gender


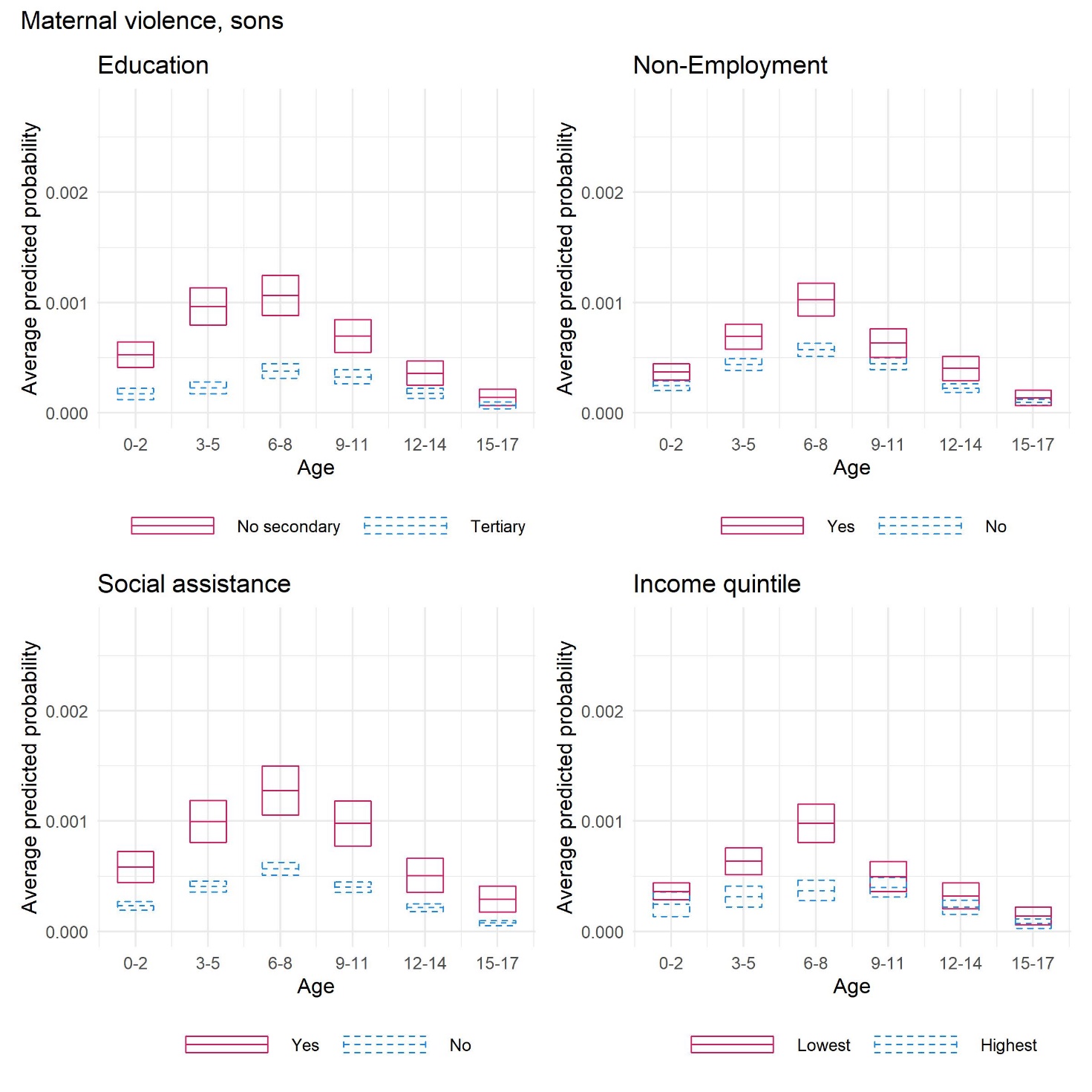


Figure 2 Predicted probabilities of violent victimization by mothers from interaction analyses between maternal SEP and child’s age. Boys.


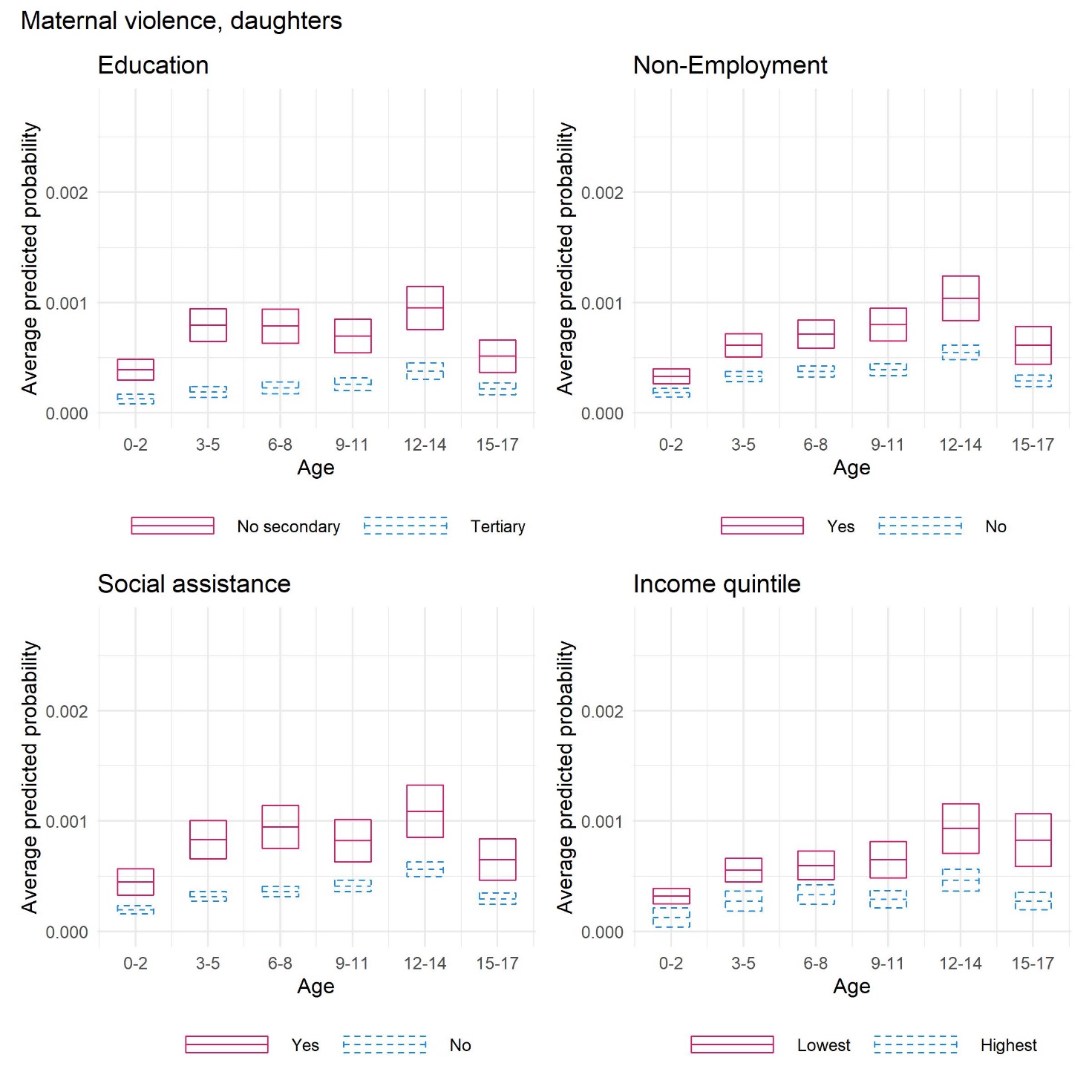


Figure 3 Predicted probabilities of violent victimization by mothers from interaction analyses between maternal SEP and child’s age. Girls.


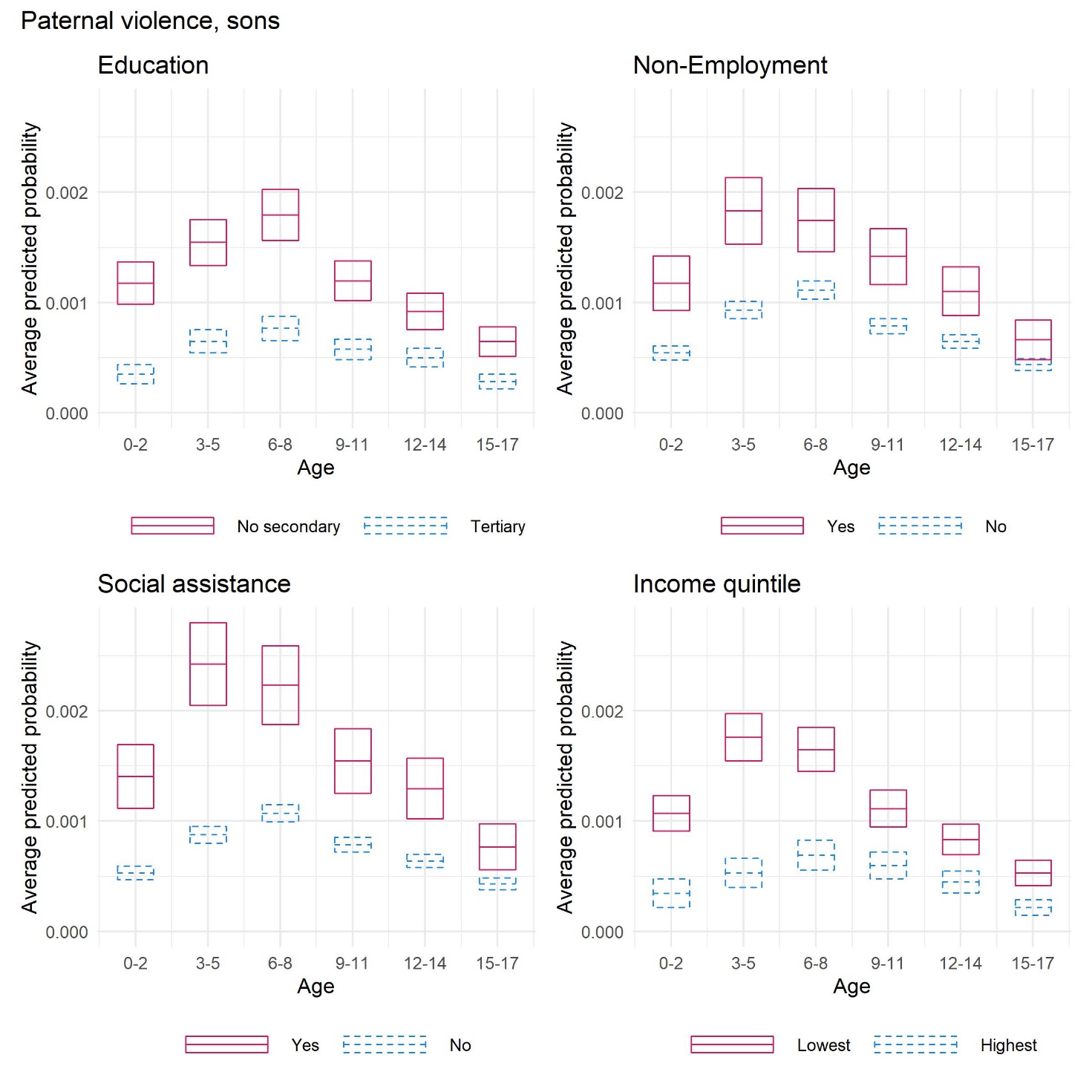


Figure 4 Predicted probabilities of violent victimization by fathers from interaction analyses between paternal SEP and child’s age. Boys.


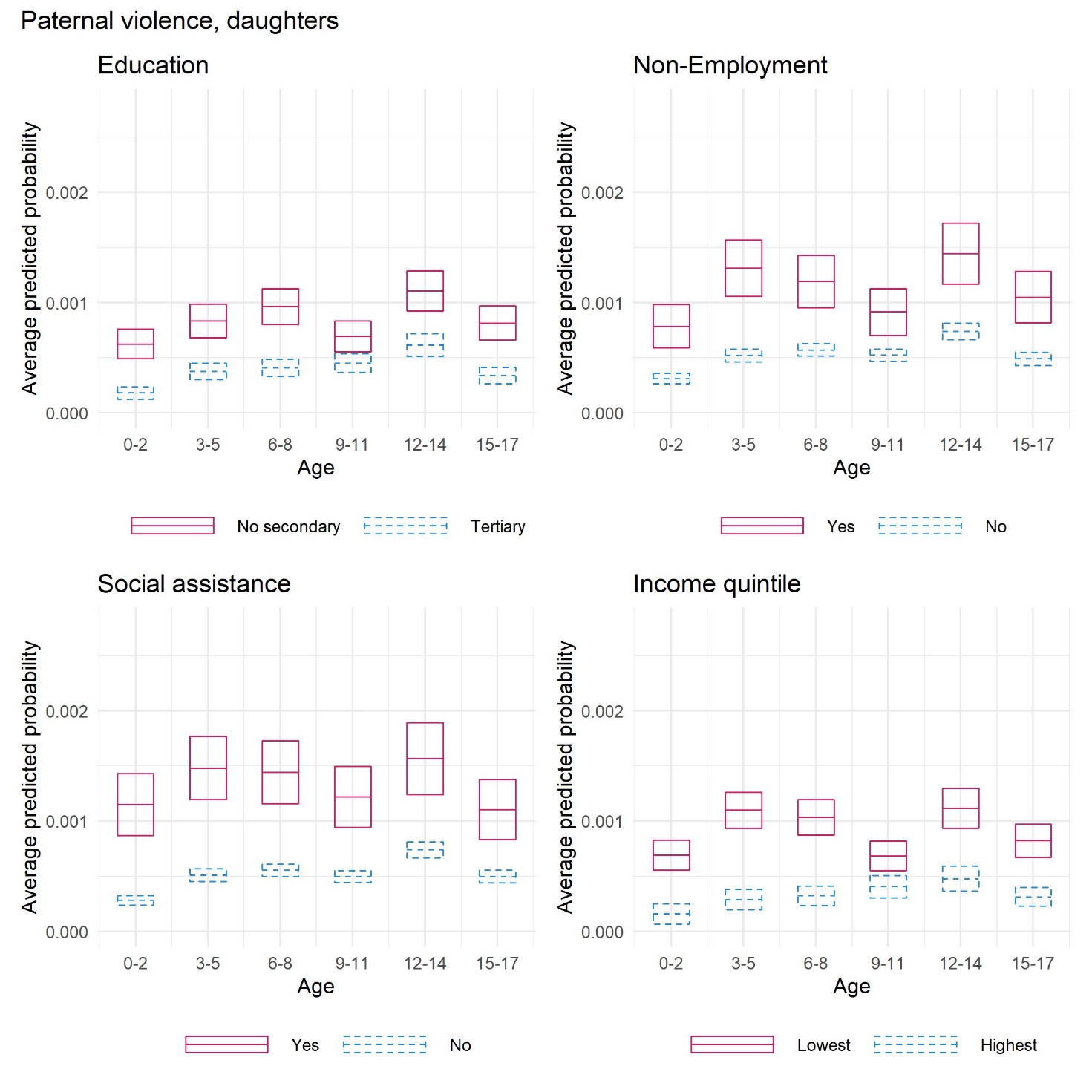


Figure 5 Predicted probabilities of violent victimization by mothers from interaction analyses between paternal SEP and child’s age. Girls.
